# Implications of Conflicting Associations of the Prion Protein (PrP) Gene with Scrapie Susceptibility and Fitness on the Persistence of Scrapie

**DOI:** 10.1371/journal.pone.0007970

**Published:** 2009-11-24

**Authors:** Andrea Doeschl-Wilson, Rami Sawalha, Simon Gubbins, Beatriz Villanueva

**Affiliations:** 1 Scottish Agricultural College, Sustainable Livestock Systems, Edinburgh, United Kingdom; 2 Institute for Animal Health, Pirbright Laboratories, Pirbright, Surrey, United Kingdom; 3 Departamento de Mejora Genética Animal, Instituto Nacional de Investigación y Tecnología Agraria y Alimentaria (SGIT-INIA), Ministerio de Ciencia e Innovación, Madrid, Spain; University of Liverpool, United Kingdom

## Abstract

**Background:**

Existing mathematical models for scrapie dynamics in sheep populations assume that the PrP gene is only associated with scrapie susceptibility and with no other fitness related traits. This assumption contrasts recent findings of PrP gene associations with post-natal lamb survival in scrapie free Scottish Blackface populations. Lambs with scrapie resistant genotypes were found to have significantly lower survival rates than those with susceptible genotypes. The present study aimed to investigate how these conflicting PrP gene associations may affect the dynamic patterns of PrP haplotype frequencies and disease prevalence.

**Methodology/Principal Findings:**

A deterministic mathematical model was developed to explore how the associations between PrP genotype and both scrapie susceptibility and postnatal lamb mortality affect the prevalence of scrapie and the associated change in PrP gene frequencies in a closed flock of sheep. The model incorporates empirical evidence on epidemiological and biological characteristics of scrapie and on mortality rates induced by causes other than scrapie. The model results indicate that unfavorable associations of the scrapie resistant PrP haplotypes with post-natal lamb mortality, if sufficiently strong, can increase scrapie prevalence during an epidemic, and result in scrapie persisting in the population. The range of model parameters, for which such effects were observed, is realistic but relatively narrow.

**Conclusions/Significance:**

The results of the present model suggest that for most parameter combinations an unfavourable association between PrP genotype and post-natal lamb mortality does not greatly alter the dynamics of scrapie and, hence, would not have an adverse impact on a breeding programme. There were, however, a range of scenarios, narrow, but realistic, in which such an unfavourable association resulted in an increased prevalence and in the persistence of infection. Consequently, associations between PrP genotypes and fitness traits should be taken into account when designing future models and breeding programmes.

## Introduction

Incidences of scrapie, a fatal transmissible spongiform encephalopathy (TSE) of sheep and goats, have been reported in European countries for several centuries [1], but progress on the control of this disease was long inhibited by the restricted understanding of the causes of infection and modes of transmission. The discovery that polymorphisms at codons 136, 154 and 171 of the prion protein (PrP) gene determine susceptibility to classical scrapie [2]–[4], constituted a major break-through for scrapie control. Since then national breeding programmes aimed to reduce and eventually eradicate small ruminant TSEs have been established in several European countries.

The scrapie eradication policies were strongly influenced by the predictions of mathematical models, which incorporated emerging information on host genetics and scrapie epidemiology and predicted the impact of selection on changes in PrP gene frequencies and scrapie prevalence over time (e.g. [5], [6]). Although the numerous mathematical models and policies vary widely in their approach and underlying assumptions, they unanimously build upon the assumption that polymorphisms of the PrP gene are associated only with scrapie susceptibility and not with any other trait such as fitness or performance. Under this assumption, models predict that scrapie will eventually disappear from a closed population as a result of natural selection (e.g. [7], [8]). The apparent conflict of these predictions with the fact that scrapie has been persistent over centuries has been explained by the exceptionally long time scales inherent in scrapie epidemiology [9].

Existing scrapie models may be challenged by recent findings in scrapie free flocks of Scottish Blackface sheep, for which an association of the PrP genotype, defined by polymorphisms at codons 154 and 171, with postnatal lamb survival has been identified [10]. The presence of the ARQ (scrapie susceptible) haplotype was generally associated with lower postnatal lamb mortality, while the presence of two ARR (scrapie resistant) haplotypes was mostly associated with increased lamb mortality rate. An association of susceptible haplotypes to lower mortality rates, may influence not only the changes of haplotype frequencies over time, but also the severity and duration of scrapie epidemics. In particular, it may have consequences on the long-term persistence of scrapie.

In this study a deterministic partial differential equations model was developed to explore how the associations between PrP genotype and both scrapie susceptibility and postnatal lamb mortality affect the prevalence of scrapie and the associated change in PrP gene frequencies in a closed flock of sheep. It will be demonstrated that this association can have strong implications for the persistence of scrapie.

## Materials and Methods

### Assumptions

Sheep are characterised by their scrapie infection status (infected or not infected), age and PrP genotype. It is assumed that the PrP gene is associated with susceptibility to scrapie and with lamb mortality from birth to 180 days of age caused by events other than scrapie (hereafter denoted non-scrapie lamb mortality). It is further assumed that the association between PrP genotype and lamb mortality is due to pleiotropy rather than linkage, implying that the associations remains constant over successive generations. For simplicity, it is further assumed that there are only two haplotype variants at the PrP locus: SL (associated with scrapie susceptibility and low non-scrapie lamb mortality) and RH (associated with scrapie resistance and high non-scrapie lamb mortality). Further, given the evidence that the risk of infection is greatest during the perinatal period and decreases with age [11], [12], it is assumed that scrapie infection occurs exclusively at or close to birth and that scrapie infected animals remain in the population until clinical signs appear. After the onset of clinical disease animals no longer remain in the flock (through death or removal). The model represents one closed flock with an initial frequency of the SL haplotype of *p_0_*. It is assumed that the flock size remains constant, i.e. animals that disappear from the population, whether due to scrapie or other causes, are replaced immediately by newborn animals.

### Population dynamics

Let *N_i_*(*a*, *t*) and *X_i_*(*a*, *t*) denote, respectively, the total number of animals and the number of infected animals of genotype *i*, of age *a* at time *t*. The change of *N* and *X* with respect to age and time is described by:
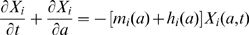


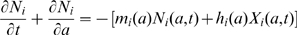
where *m_i_*(*a*) is the non-scrapie mortality rate for genotype *i* (SL/SL, SL/RH or RH/RH) at age *a*, and *h_i_*(*a*) is the hazard function for the log-normal age-at-onset of scrapie distribution with genotype specific parameters *μ_i_* and *σ_i_* [6], representing death due to scrapie.

The PrP genotype specific non-scrapie mortality rate is:
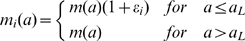
where *m*(*a*) is the age-dependent baseline non-scrapie mortality rate, which is equal for all PrP genotypes, and *ε*_i_ is the factor by which mortality increases for genotype *i* for ages below a certain threshold *a_L_*. The baseline rate *m*(*a*) was determined using a Weibull distributed survivorship curve for the average mortality rate of the three PrP genotypes, i.e. 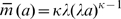 [7].

The prevalence of infection at time *t*, *P*(*t*), is then calculated as the proportion of infected animals in the population, i.e.
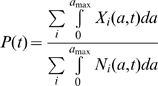
where *a*_max_ refers to the maximum age in the population, which is defined by the survivorship curve described above.

### Boundary conditions and initial conditions (*a* = 0 and t = 0)

The number of infected newborns of genotype *i* is calculated following the approach of Gubbins and Roden [6], relating the proportion of newly infecteds to the current population prevalence and assuming that all animals of a given genotype experience the same force of infection, yielding
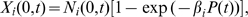
(1)where *β_i_* is a genotype specific transmission parameter. The number of newborns of genotype *i* at time *t* is given by
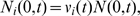
where *ν_i_*(*t*) denotes the proportion of genotype *i* animals in the newly born population of size *N*(0,*t*) at time *t*. *N*(0,*t*) is calculated such that the overall population size remains steady by setting the birth rate equal to the total death rate and *ν_i_*(*t*) is calculated from the haplotype frequency of the breeding parents, which comprise all animals within the fertile age range [*a_F1_*, *a_F2_*]. Assuming random mating and constant prolificacy across the fertile period, the frequencies of SL/SL, SL/RH and RH/RH are *p* ^2^, 2*p*(1−*p*) and (1−*p*)^2^, respectively, where *p* is the frequency of haplotype SL in the breeding population at time *t*.

The initial genotype and age distribution *N_i_*(*a*,0) is determined by the initial frequency of the SL haplotype *p_0_* and the Weibull-distributed survivorship curve. Scrapie is introduced by *X_i_*(*a_0_*,0) infected individuals of genotype i with age *a_0_*.

### Parameter values and sensitivity analysis

Table 1 lists the model parameters and their value ranges, together with the corresponding empirical estimates, considered in this study. Baseline values for the genotype specific model parameters *μ_i_*, *σ_i_*, *β_i_* and *ε_i_*, were derived from existing empirical estimates for genotypes ARQ/ARQ, ARQ/ARR and ARR/ARR, which represent respectively genotypes SL/SL, SL/RH and RH/RH in our model. Based on the empirical evidence and to facilitate the understanding of the key factors behind a specific model behaviour, the transmission parameter *β*_2_ and the hazard rate parameters (*μ*_2_, *σ*_2_) corresponding to the heterozygote were initially set to zero, implying that only the homozygote SL/SL is susceptible to scrapie. Likewise, the non-scrapie lamb mortality coefficient *ε*_2_ was initially set to zero implying that only the homozygote RH/RH has increased lamb mortality compared to the other PrP genotypes.

**Table 1:** Values for model parameters and empirical estimates.

Parameter	Description	Value (range) used(a)	Source/empirical estimate(b)
*β_i_*, *i* = 1,2,3	Scrapie transmission parameter for genotype *i*		Gubbins & Roden [6]:
		*β*_1_ = 2.0–10.0	*β*_1_ = 2.8–4.93
		*β*_2_ = 0(c)	*β*_2_ = 0.02–0.04
		*β*_3_ = 0	*β*_3_ = 0
*ε_i_*, i = 1,2,3	Discrepancy in non-scrapie lamb mortality rate for genotype *i* from average age specific mortality rate		Sawalha et al. [10]
		*ε*_1_ = 0	*ε*_1_ = 0.0065
		*ε*_2_ = 0.0(c)	*ε*_2_ = 0.0
		*ε*_3_ = 0.0–0.1	*ε*_3_ = 0.0225
(*μ_i_*, *σ_i_*)	Mean and standard deviation for the age-at-onset distribution *h_i_(a)*		Gubbins & Roden [6]
		(μ_1_, σ_1_) = (1.20, 0.43)	(*μ*_1_, *σ*_1_) = (1.20, 0.43)
		(μ_2_, σ_2_) = (0,0)(c)	(*μ*_2_, *σ*_2_) = (1.54, 0.38)
		(μ_3_, σ_3_) = (0,0)	(*μ*_3_, *σ*_3_) = (0,0)
*p*_0_	Initial frequency of the susceptible haplotype SL	*p*_0_ = 0.0–1.0	Arbitrary
(*κ*, *λ*)	Parameters for Weibull-survivorship function	*κ* = 2, *λ* = 0.2216(d)	Stringer et al. [7]
*a_L_*	Max. age with genotype dependent mortality rate	*a_L_* = 180 days	Sawalha et al. [6]
[*a_F1_*, *a_F2_*]	Fertile age range	*a_F1_* = 6 months, *a_F2_* = 6 years	Lewis & Simm [13]
*a_0_*	Age at first infection	*a_0_* = 0.5–4 years	Arbitrary
*X*_1_(*a*_0_, 0)/*N*_1_(*a*_0_, 0)	Proportion of initially infected animals	0.0001	Arbitrary

(a) Subscripts *i* = 1,2,3 refer to the genotypes SL/SL, SL/RH and RH/RH, respectively.

(b) Subscripts *i* = 1,2,3 refer to the genotypes ARQ/ARQ, ARQ/ARR and ARR/ARR, respectively.

(c) The values in the table refer to the results presented in this paper. Model results were also generated for (*μ*_2_, *σ*_2_) = (1.54, 0.38), *β*_2_ = 2.0–10.0 (*β*_2_≤*β*_1_), *ε*_2_ = 0.0–1.0 (ε_2_≤ε_3_).

(d) Corresponds to an average life expectancy of 4 years.

Empirical estimates exist also for the parameters specifying the average non-scrapie mortality rate (*κ*, *λ*), the fertile age range [*a_F1_*, *a_F2_*], and the maximum lamb age *a_L_* for which different PrP genotypes have different mortality rates (Table 1). The remaining model parameters (*p_0_* and *a_0_*) were assigned arbitrary values.

The influence of each model parameter was investigated using sensitivity analysis, in which model predictions were obtained by varying either one parameter at a time or a combination of parameters. Parameter ranges used in the simulations were either 95% upper and lower confidence limits of the estimates obtained in the original studies (Table 1) if those were available, or parameter ranges that were biologically realistic. Identified critical parameters that significantly altered the trends of scrapie prevalence and haplotype frequencies were *β*_i_, *ε*_i_, and *p_0_*. The duration of the incubation period, as specified by the hazard function parameters (*μ*_i_, *σ*_i_) was also found to have a significant influence on the model predictions. However, similar patterns for changes in scrapie prevalence and haplotype frequencies as those observed for different values *μ*_i_ and *σ*_i_ were obtained by modifying the transmission rates. Therefore, the hazard function was kept fixed, and the results presented show the model behaviour for a biologically realistic range of values of the parameters *β_i_*, *ε_i_* and *p_0_* (Table 1).

### Prevalence threshold

Since the model is continuous, the predicted scrapie prevalence can take values that, although numerically different to zero, correspond to less than one infected individual. For the results presented here it was assumed that outbreaks resulting in prevalence values corresponding to less than one infected sheep would result in a stochastic fade-out of the disease. The population size simulated was *N* = 100,000 and therefore the prevalence threshold below which scrapie was considered eradicated was 0.001%.

## Results

**Figure 1:**
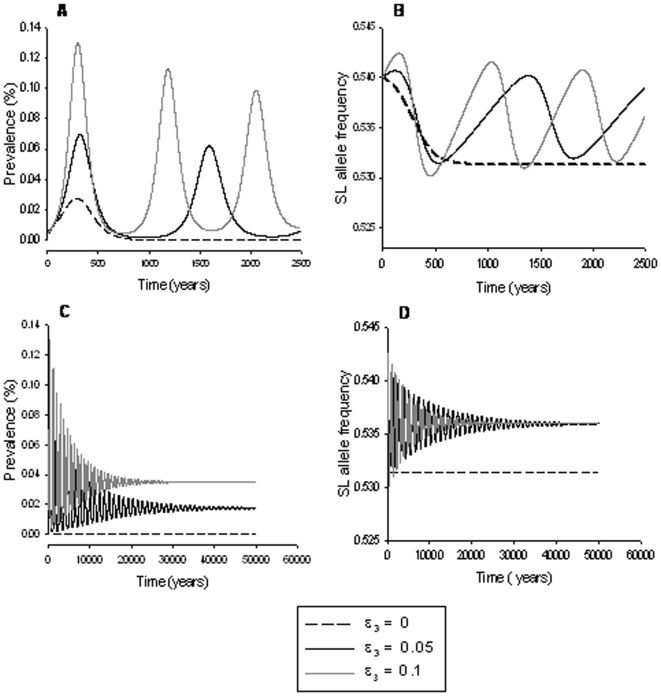
Predicted scrapie prevalence and frequency of the susceptible SL haplotype for different values of the parameter ε_3_. Top graphs show predictions for 2500 years, whereas the bottom graphs illustrate the behavior up to the endemic equilibrium. Other parameter choices are *ε*_1_ = *ε*_2_ = 0, *p*_0_ = 0.54, *β*_1_ = 4.93 and *β*_2_ = *β*_3_ = 0. For description of the parameters and other parameter values see table 1.

Assuming first that the PrP genotype is only associated with scrapie susceptibility (i.e. *ε*_i_ = 0 for all *i*), so that changes in PrP haplotype frequencies are exclusively caused by scrapie related mortality, our model predicts that scrapie eventually disappears from the population within a few decades or centuries (Figures 1a, 1c). The frequency of the susceptible haplotype declines towards a non-zero steady state value (Figure 1d). This behaviour was observed for all parameter combinations within the admissible range shown in Table 1. Inclusion of unfavourable associations between the scrapie resistant PrP haplotype and non-scrapie lamb mortality (i.e. *ε*_3_>0) does not drastically alter this behaviour for the majority of model parameter values. The lower average life expectancy and prolificacy of scrapie susceptible genotypes remain the main drivers for frequency changes throughout the epidemic phase of the infection, and lead to the gradual decrease of susceptible genotypes until the disease can no longer be sustained. The additional boost in the replenishment of susceptible genotypes due to their lower mortality rates is too weak to prevent scrapie from gradually dying out.

**Figure 2:**
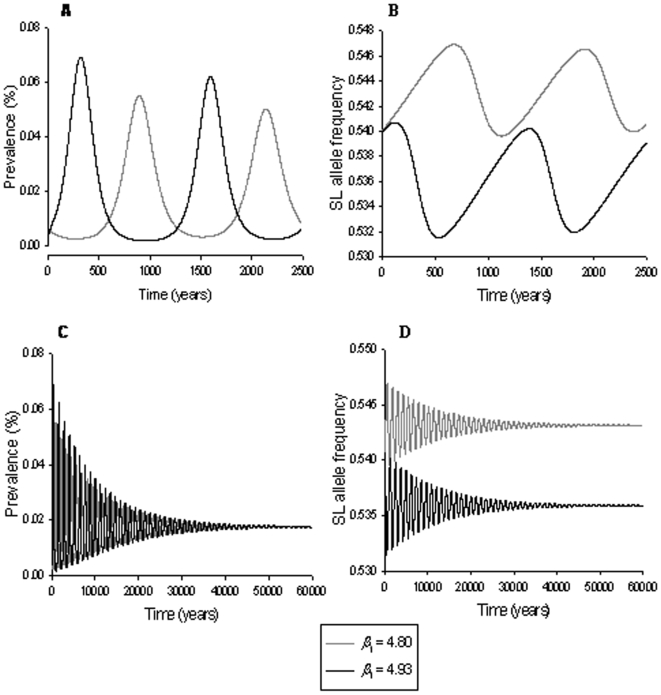
Predicted scrapie prevalence (left) and frequency of the SL haplotype (right) for different values of the parameter *β*_1_. Top graphs show predictions for 2500 years, whereas the bottom graphs illustrate the behavior up to the endemic equilibrium. Other parameter choices are *ε*_1_ = *ε*_2_ = 0, *ε*_3_ = 0.05, *p*_0_ = 0.54, and *β*_2_ = *β*_3_ = 0. For description of the parameters and other parameter values see table 1. Long-term persistence was only observed for 4.80≤*β*_1_≤4.93.

**Figure 3:**
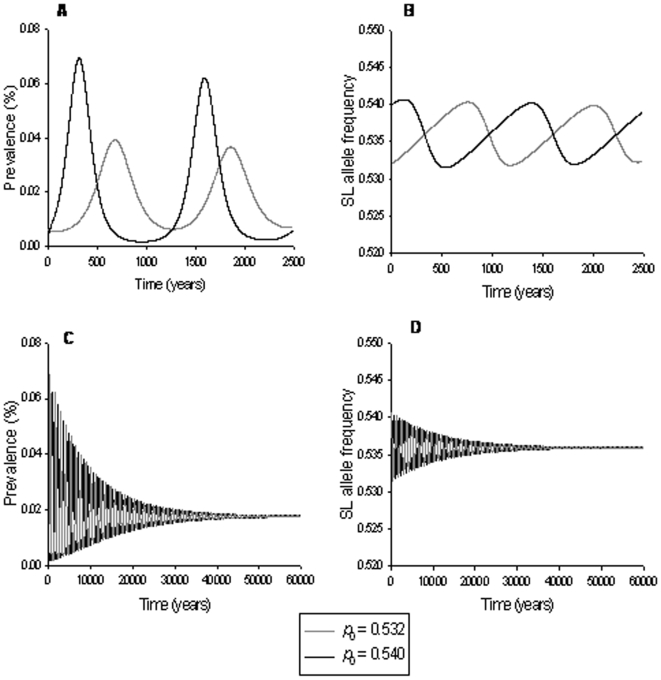
Predicted scrapie prevalence (left) and frequency of the SL haplotype (right) for different values of the parameter *p*_0_. Top graphs show predictions for 2500 years, whereas the bottom graphs illustrate the behavior up to the endemic equilibrium. Other parameter choices are *ε*_1_ = *ε*_2_ = 0, *ε*_3_ = 0.05, *β*_1_ = 4.93 and *β*_2_ = *β*_3_ = 0. For description of the parameters and other parameter values see table 1. Long-term persistence was only observed for 0.532≤*p_0_*≤0.540.

However, for some parameter values within the admissible range, the association of PrP genotype with non-scrapie lamb mortality causes scrapie to prevail in the population over much longer timescales (Figures 1, 2, 3). Whether scrapie is expected to persist depends mainly on the combination of the strength of association of PrP genotype with non-scrapie lamb mortality (*ε*_i_), the disease transmission rate (*β*_i_) and the initial proportion of susceptible haplotypes (*p_0_*). Sufficiently high values for *ε*_3_ are a prerequisite for the long-term persistence of scrapie. Extensive search through the model parameter space produced only long-term scrapie persistence for *ε*_3_≥0.01. It was also found that long-term persistence is only associated with combinations of *p*_0_ and *β*_1_ that correspond to weak prevalence levels at all times (generally less than 0.2% for 0≤*ε*_3_≤0.1). Combinations of *p*_0_ and *β*_1_ resulting in more severe initial outbreaks also produce a rapid decline in the proportion of susceptible sheep to a level that is insufficient for sustaining the disease.

Table 2 shows, for given values of *β*_1_ and *ε*_3_, the range of *p*_0_ that lead to persistent scrapie prevalence above the threshold 0.001%. When *β*_1_ is low (i.e. 2. 0), scrapie persists only when *p_0_* is relatively high (∼84.5%). In contrast, higher *β*_1_ requires a lower *p_0_* for preventing scrapie to disappear. The stronger the association of PrP genotype with non-scrapie lamb mortality (i.e. the higher *ε*_3_) the wider the range of *p*_0_ values corresponding to long-term persistence of the disease.

**Table 2:** Range of the initial frequency of the susceptible SL haplotype (*p*_0_) corresponding to long-term persistence of scrapie for different values of the scrapie transmission parameter *β*_1_ and the non-scrapie mortality rate coefficient *ε*_3_.

*ε*_3_ (a)	range of *p_0_*			
	*β*_1_ = 2.00	*β*_1_ = 2.80(b)	*β*_1_ = 4.93(b)	*β*_1_ = 10.00
0.01	0.845–0.845	0.712–0.712	0.535–0.536	0.376–0.377
0.02	0.844–0.845	0.710–0.714	0.533–0.539	0.375–0.379
0.05	0.843–0.846	0.709–0.716	0.532–0.540	0.373–0.381
0.08	0.842–0.847	0.708–0.717	0.531–0.542	0.372–0.383
0.10	0.842–0.848	0.707–0.719	0.530–0.544	0.371–0.384

(a) No long-term persistence was observed for *ε*_3_<0.01.

(b) The values *β*_1_ = 2.80 and *β*_1_ = 4.93 were the lower and upper bound estimates of Gubbins and Roden [6].

For a full description of the parameters and of other parameter values used see table 1.

As illustrated in Figures 1, 2 and 3, long-term persistence of scrapie is characterised by a damped oscillatory behaviour of both scrapie prevalence and SL haplotype frequency, which converge eventually to endemic equilibrium states. All parameter combinations that do not result in scrapie fading out immediately after the introduction of an infected sheep or after the first outbreak produce similar long-term persistence patterns.

Figures 1, 2 and 3 also depict the influence of the key parameters *ε*_3_, *β*_1_ and *p*_0_, on the amplitudes and frequencies of the oscillations and on the equilibrium states. Stronger associations between PrP genotype and non-scrapie lamb mortality (i.e. higher values of *ε*_3_) generate oscillations in both scrapie prevalence and SL haplotype frequency of higher amplitudes and higher frequency (Figures 1a, 1b). They also generate stronger damping and lead to higher predicted disease prevalence at the endemic equilibrium (Figure 1c). The steady state SL frequency corresponding to *ε*_3_>0 is higher than that corresponding to *ε*_3_ = 0, but is the same for different non-zero *ε*_3_ values (Figure 1d).

The range of values for the transmission parameter *β*_1_ leading to scrapie persistence for fixed values of the other model parameters is narrow. For example, as shown in Figure 2 for *p*_0_ = 0.54 and *ε*_3_ = 0.05, scrapie is only predicted to persist when 4.80≤*β*_1_≤4.93. Values of *β*_1_ outside this range result in scrapie fading out either immediately after the introduction of the disease (*β*_1_<4.80) or in a too high mortality of susceptible genotypes to maintain a scrapie prevalence above the required threshold after the first outbreak (*β*_1_>4.93). The persistence patterns of scrapie prevalence and SL frequency are also sensitive to changes in *β*_1_. For example, decreasing the transmission rate may cause a delay in the initial outbreak (scrapie prevalence only starts to rise substantially after sufficient SL haplotypes are available in the population, Figures 2a, 2b) and result in a higher steady state SL haplotype frequency (Figure 2d). The steady state prevalence is however the same for different values of *β*_1_ corresponding to long-term scrapie persistence (Figure 2c).

The amplitudes of the oscillations in scrapie prevalence and haplotype frequency were also found highly sensitive to changes in the initial SL frequency *p*_0_ (Figure 3). Similar to the effect of high transmission parameter values *β*_1_, higher proportions of scrapie susceptible SL haplotypes in the population lead to faster and more severe outbreaks with consecutively stronger decline in disease prevalence (Figure 3a). Slight increases in the proportion of susceptible haplotypes were found to rapidly cause the prevalence to fall below the assumed persistence threshold of 0.001% after the first scrapie outbreak. The long-term patterns of scrapie prevalence and SL haplotype frequency, including the corresponding equilibrium states were however found to be independent of *p*_0_ (Figures 3c, 3d).

## Discussion

In this study a model was developed to determine the influence of conflicting associations of the PrP genotype with scrapie susceptibility and lamb mortality on PrP haplotype frequencies and scrapie prevalence patterns. The potential impact of such associations on scrapie epidemiology and PrP haplotype frequencies had been drawn to attention in previous studies [9], [14], but was dismissed due to lack of empirical evidence. Recent findings of a positive association of the scrapie susceptible ARQ haplotype with post-natal lamb survival rate in scrapie free Scottish Blackface flocks [10] however suggest that the earlier speculations merit further consideration. Based on a quantitative genetics approach, the conflicting PrP genotype associations with respect to scrapie susceptibility and fitness imply that the scrapie susceptible haplotype will forever prevail in the population at high frequency (approx. 0.9, see Figure S1 in the supplementary material). These results may warrant speculations that scrapie is also likely to persist in the population without human intervention [10]. Quantitative predictions on the long-term persistence of scrapie require however also the consideration of epidemiological disease characteristics.

The model presented here captures relevant genetic and epidemiological aspects of scrapie and implements estimates derived from real data as baseline values for the model parameters. Results show that for the majority of model parameter combinations, associations of the PrP gene with lamb mortality in addition to scrapie susceptibility have little impact on the persistence patterns of scrapie. Only under very specific conditions, the associations between PrP genotype and lamb mortality may lead to scrapie persisting in sheep populations in the absence of human intervention. The range of model parameters leading to persistence of scrapie is narrow, but realistic. Prevalence patterns have similar characteristics to those observed in real populations, manifesting themselves in prolonged, but relatively mild recurring outbreaks, with a small percentage of sheep infected at any time. They eventually settle into an endemic equilibrium of low prevalence.

These results, although in line with empirical observations, contrast those of previous modelling studies that assume no PrP gene association with other traits besides scrapie. Using a genetic-epidemiological model that model that relates PrP genotypes only to scrapie susceptibility, Woolhouse et al. [8] predicted that scrapie will be eliminated within decades or centuries in closed flocks without human intervention. The same behaviour was observed in our model when PrP genotypes were assumed only to be related to scrapie susceptibility (i.e. setting ε_3_ to zero). However, our model shows that the predicted scrapie dynamics can be altered when considering conflicting associations of the PrP genotype with fitness traits other than scrapie susceptibility, even if they are weak. In particular, favourable associations of the PrP susceptible genotype with fitness can stimulate the replenishment of susceptible animals to a level that is sufficient to prevent scrapie from disappearing.

The conditions for long-term persistence of scrapie in our model are determined by specific combinations of the disease transmission parameter, the PrP genotype specific increase in non-scrapie mortality rate and the PrP haplotype frequencies at the onset of the outbreak (Table 2). Parameter combinations that cause scrapie outbreaks with peak prevalences exceeding approximately 0.2% in our model also wipe out the required proportion of susceptible animals for maintaining the disease without re-introduction of the infection. A more accurate representation of flock structure and transmission patterns would likely increase this threshold.

There are several reasons that explain why the range of model parameters leading to long-term persistence of scrapie is narrow. First, the transmission parameter in this model occurs in the exponent of an exponential function (equation 1), and model outputs are highly sensitive to changes of the exponent. Representing the transmission of scrapie in this way had the advantage that only one parameter (i.e. *β*) was needed to describe the currently still largely unknown transmission process. However, if disease incidence was assumed to depend linearly on disease transmission rates, as in standard SIR models, a lower sensitivity of the model results with respect to changes in *β* would be expected and similar prevalence patterns would be observed over a wider parameter range. Second, the threshold for scrapie prevalence corresponding to one infected individual implies that the range of model parameters corresponding to long-term persistence of scrapie in this model is partly dependent on the population size. The simulated population comprised 100,000 animals. A larger population with the same initial prevalence at the onset of infection would result in identical predictions for PrP haplotype frequencies and scrapie prevalence, but would also impose a lower prevalence threshold corresponding to one infected individual. Consequently the range of model parameters producing prevalence consistently above the imposed threshold would be wider for larger population sizes.

In this study only two haplotype variants (SL and RH) were assumed to exist at the PrP locus. The haplotypes may represent the ARQ and ARR haplotypes, which are the most frequent haplotypes in UK Scottish Blackface sheep, and which were shown to be linked to lamb mortality in the study of Sawalha et al. [10]. Results presented here are also based on the assumption that only SL/SL genotypes are susceptible to scrapie and that only RH/RH genotypes have higher lamb mortality rates not related to scrapie infections. However, to test the broader implications of our results, simulations carried out with different dominance assumptions (i.e. SL/RH genotypes are also susceptible to scrapie and have different lamb mortality rates than SL/SL genotypes) resulted in similar long-term scrapie persistence patterns, albeit for different ranges of model parameters.

Numerous recent studies have investigated the relationship between PrP genotype and performance traits (e.g. [15]–[17]). Whereas accumulative evidence suggests that there is no consistent association between PrP genotype and performance, evidence for the association with fitness traits is still sparse (as reviewed by [18]). In a recent study Gubbins et al. [19] investigated associations between PrP genotype and post-natal lamb mortality for ten sheep breeds in the UK, including the Scottish Blackface. They found no significant associations for a majority of breeds, although the observed trend for Scottish Blackface was similar to that found by Sawalha et al. [10]. The discrepancies in the results of both studies could be due to differences in the datasets – the analysis of Gubbins et al. [19] was based on commercial data in contrast to the higher number and more reliable research data used by Sawalha et al. [10]. The discrepancies could however also point to breed-specific associations between the PrP gene and genes controlling survival due to linkage disequilibrium. The consequence of this for our model is that the linkage could break down over generations, weakening or eliminating the associations between PrP genotype and lamb survival. This would result in a reduced risk of scrapie persisting in the population. Our model simulates the worst case scenario, where associations are assumed to be due to pleiotropy rather than linkage or where the linkage disequilibrium is maintained over many generations.

The PrP genotype was assumed to be only associated with scrapie susceptibility and post-natal lamb mortality in our model. However, given that a number of genes in close proximity to the PrP gene on chromosome 13 are involved in immune response functions, it is possible that additional associations of the PrP genotype with other health or fitness traits will be identified in the future [18]. Selective advantage for genotypes that are susceptible to classical scrapie may also occur as a result of different strains of scrapie in the population affecting different PrP genotypes, as outlined by Slate [14] and supported by recently emerging evidence for atypical scrapie that occurs more frequently in sheep with haplotypes resistant to classical scrapie (e.g. ARR) [20], [21]. The results of the present model support the arguments of Slate [14] that these associations should be taken into account when designing future models and breeding programmes.

For the design and evaluation of breeding programs more accurate representations of the structure of existing sheep populations and of scrapie biology would be required. For example, as demonstrated in various previous modelling studies, prevalence patterns are strongly influenced by (i) the representation of the transmission processes in the model (e.g. distinction between horizontal and vertical disease transmission scenarios) (e.g. [7], [22], [23]), (ii) the stratification of the sheep population in individual flocks of different sizes with sex specific reproduction rates, and transmission occurring both within and between flocks [24]–[26], (iii) the pathogenesis of scrapie [8], [23], (iv) seasonal patterns in the population structure [23], as well as (v) the distribution of breeds and PrP genotypes across the region under consideration [5], [27]. The model presented here could be extended to account for all these factors.

## Supporting Information

Text S1 A quantitative geneticist's approach for calculating change in allele frequency
(0.03 MB DOC)

Figure S1 Expected change of the SL allele frequency over successive generations for different initial allele frequencies (p_0_) assuming values for coefficients of selection s_1_ (associated with scrapie susceptibility) and s_2_ (associated with PrP specific increase in lamb mortality) of 0.0022 and 0.02, respectively.
(0.03 MB TIF)
