# Supplementary material for: Implications of Conflicting Associations of the Prion Protein (PrP) Gene with Scrapie Susceptibility and Fitness on the Persistence of Scrapie
Source: PLoS One. 2009 Nov 24;4(11):e7970. doi: 10.1371/journal.pone.0007970 (PMC2776355; doi:10.1371/journal.pone.0007970)
Supplement: Text S1 — A quantitative geneticist's approach for calculating change in allele frequency (0.03 MB DOC) [file pone.0007970.s001.doc]

**Electronic supplementary material:**

**A quantitative geneticist’s approach for calculating change in allele frequency**

Falconer and MacKay ([28], chapter 2) provide a simple framework for estimating changes in allele frequencies brought upon by the PrP gene associations with scrapie susceptibility and lamb mortality, which is based on the calculation of gamete contributions of one generation toward their offspring. In quantitative genetics terms, these two associations imply that selection acts (1) against the genotypes that are susceptible to scrapie as well as (ii) against the genotypes with increased lamb mortality.

Assume that the allele RH is completely dominant for scrapie resistance and completely recessive for the PrP gene associated increase in lamb mortality. Relative to the heterozygote RH/SL, the scrapie susceptible homozygote SL has a coefficient of selection, i.e. a relative reduction in gamete contribution, of s_1_ (0 < s_1_<1) and the homozygote RH/RH has a coefficient of selection s_2_ (0 < s_2_<1) due to its increased lamb mortality rate (Table S1). Let p_n_ be the frequency of allele SL at generation n. Then summing the gamete contributions of individual genotypes (Table S1), the total gamete contribution of generation n towards generation n+1 is 1-s_2_(1-p_n_)^2^-s_1_p_n_^2^. .

**Table S1.** Frequencies, coefficients of selection and gamete contributions for the three genotypes

|  | RH/RH | RH/SL | SL/SL |
| --- | --- | --- | --- |
| Frequency at generation n | (1-p_n_)^2^ | 2(1-p_n_)p_n_ | p_n_^2^ |
| Coefficient of selection | s_2_ | 1 | s_1_ |
| Gamete contribution | (1- s_2_)(1-p_n_)^2^ | 2(1-p_n_)p_n_ | (1- s_1_) p_n_^2^ |

The frequency of the susceptible allele in generation n+1 is the gamete contribution of genotype SL/SL plus half of the gamete contribution of genotype RH/SL relative to the total, i.e.

$p_{n+1}=\frac{(1-s_{1})p_{n}^{2}+(1-p_{n})p_{n}}{1-s_{2}(1-p_{n})^{2}-s_{1}p_{n}^{2}}=p_{n}\frac{1-s_{1}p_{n}}{1-s_{2}(1-p_{n})^{2}-s_{1}p_{n}^{2}}$ (1)

Equation (1) provides the conditions for changes in allele frequency. In particular, allele frequencies are stable if p_n+1_ = p_n_, i.e. if

$\frac{1-s_{1}p_{n}}{1-s_{2}(1-p_{n})^{2}-s_{1}p_{n}^{2}}=1$, (2)

which, after solving the above equation for p_n_, leads to the necessary condition:

p_n_ =1 or p_n_ = s_2_/(s_2_+s_1_).

Similarly, the frequency of the scrapie susceptible allele SL increases from one generation to the next (i.e. p_n+1_ > p_n_) if the left hand side of equation (2) is greater than 1, i.e. if

p_n_ < $\frac{s_{2}}{s_{2}+s_{1}}$,

and decreases if

$\frac{s_{2}}{s_{2}+s_{1}}<p_{n}<1$.

The above inequalities directly show that whether the frequency of the scrapie susceptible allele SL increases or decreases from one generation to the next depends on the coefficients of selection associated with scrapie (s_1_) and increased lamb mortality (s_2_), as well as on the allele frequency itself. Further, using simple algebra, it can be proven that the following inequality holds:

|p_n_ – p_n+1_| < |p_n_ - |

Thus, a population with any given initial frequency 0 < p_n_ < 1 is predicted to converge monotonically towards the equilibrium frequency p*, which is determined by the selection coefficients s_1_ and s_2_:

p* = $\frac{s_{2}}{s_{2}+s_{1}}$ (3)

If the initial allele frequency p_0_ < p*, the frequency increases towards p* over successive generations, whereas for p_0_ > p*, the frequency decreases towards p*. The rates of change in allele frequency, i.e. (p_n+1_ – p_n_) depend non-linearly on p_n_ and on the coefficients of selection (Equation 1). According to equation (3), the equilibrium frequency p* equals 0.5 in the special case of equal selection forces, i.e. s_1_ = s_2_. The SL allele frequencies converges to an equilibrium p*>0.5, if the selection force associated with PrP lamb mortality is greater than that associated with scrapie (s_2_ > s_1_), and towards p* < 0.5 if the opposite is true.

Figure S1 illustrates the change in SL allele frequency for different initial SL allele frequencies for coefficients of selection s_1_ = 0.0022 (based on the maximum likelihood estimates of scrapie prevalence in GB of Gubbins et al., [25]) and s_2_ = 0.02 (based on the estimate for PrP increased lamb mortality of Sawalha et al., [10]), which according to equation (3) yield an equilibrium frequency p* = 0.901. For different initial frequencies p_0_, the allele frequencies for each generation converge at a decreasing rate towards the equilibrium frequency p*. This high equilibrium value for the frequency of the susceptible allele may warrant speculations that scrapie is unlikely to disappear from sheep populations without human intervention [10].

## Figure Captions

**Figure S1.** Expected change of the SL allele frequency over successive generations for different initial allele frequencies (p_0_) assuming values for coefficients of selection s_1_ (associated with scrapie susceptibility) and *s*_2_ (associated with PrP specific increase in lamb mortality) of 0.0022 and 0.02, respectively.
