# Supplementary figures and images for: Implications of Conflicting Associations of the Prion Protein (PrP) Gene with Scrapie Susceptibility and Fitness on the Persistence of Scrapie
Source: PLoS One. 2009 Nov 24;4(11):e7970. doi: 10.1371/journal.pone.0007970 (PMC2776355; doi:10.1371/journal.pone.0007970)

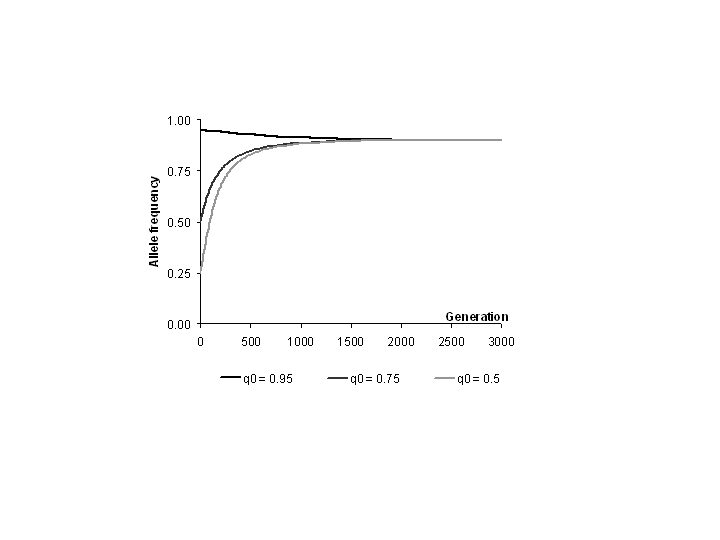

Supplement: Figure S1 — Expected change of the SL allele frequency over successive generations for different initial allele frequencies (p0) assuming values for coefficients of selection s1 (associated with scrapie susceptibility) and s2 (associated with PrP specific increase in lamb mortality) of 0.0022 and 0.02, respectively. (0.03 MB TIF) [file pone.0007970.s002.tif]
